# 

**Published:** 1890-04-19

**Authors:** 


SATURDAY, APRIL 19th, 1890.

**A Skulking Accuser** - - - - 31
**Words of Consolation.**—Death - - - 32
**Vaccination in Germany** - - - - 32
**The Doctor's Pulpit.**—A Gossip About Pills - - - 33
**The Hospital Workshop.** XXV.—The General Infirmary, Gloucester - - - - 34
**Baden-Baden** - - - - 35
**Everybody's Page** - - - - 35
**Annotations.**—Epsom College—A Gleam of Sense—Popular Errors - - - 38
**Notes and News** - - - - 36
**The Practitioner's Mirror and Retrospect:**
MEDICINE—Inebriety—Epilepsy - - - 39
SURGERY—Tracheotomy—Popliteal Aneurysm - - 39
NEW DRUGS, APPLIANCES, AND THINGS MEDICAL—Danielson and Co.'s Clinical Figures and Charts—Dee Oil Company's Preparations—Taylor's Super A1 Flax Lint - - 40
**The Sussex County Hospital, Brighton** - - 41
**Passing Topics.**—Medical Relief in London - - 42
**Scraps and Gleanings** - - - - 42

EXTRA SUPPLEMENT—THE NURSING MIRROR.

**En Passant.**—Thousands of Pounds for the National Pension Fund—South Wimbledon—Nursing in Paris—Dr. Page at Redditch—Sir Henry Acland on Nurses—Queen Victoria's Jubilee Institute—A Suggestion for a Memorial - - - ix
**Nursing Lectures.** By PERCY LEWIS, M.D. VIII.—Feeding - - - x
**The Passing of the Shadow** - - - - xi
**Presentation** - - - - xi
**Everybody's Opinion.**—Preparations for June—Nurse Training Schools - - - xii
**Appointments** - - - - xii
**Vacancies.—Notes and Queries** - - - xii
**Amusements and Relaxation** - - - xii

